# Awareness and Practice of Oral Health Measures in Medina, Saudi Arabia: An Observational Study

**DOI:** 10.3390/ijerph17239112

**Published:** 2020-12-06

**Authors:** Selma A Saadaldina, Elzahraa Eldwakhly, Ahmad A Alnazzawi, Rayan A Alharbi, Bushra K Alghamdi, Osama A Abu Hammad, Mai Soliman

**Affiliations:** 1 Schulich School of Medicine and Dentistry, Western University, London, ON N6A 3K7, Canada; ssaadal@uwo.ca; 2 Department of Fixed Prosthodontics, Faculty of Dentistry, Cairo University, Cairo 12613, Egypt; zeldwakhly@yahoo.com; 3 Clinical Dental Sciences Department, College of Dentistry, Princess Nourah Bint Abdulrahman University, Riyadh 11564, Saudi Arabia; 4 College of Dentistry, Taibah University, Medina 42353, Saudi Arabia; alnazzawi@gmail.com (A.A.A.); rayanalharbi03@gmail.com (R.A.A.); bushrah.93@gmail.com (B.K.A.); o.abuhammad@yahoo.com (O.A.A.H.); 5 School of Dentistry, University of Jordan, Amman 11942, Jordan

**Keywords:** oral health, pain, tooth brushing, oral health related behaviors, social determinants of oral health

## Abstract

The aim of this observational study is to investigate the oral health status and practices in the multicultural community of Medina, Saudi Arabia. A cross-sectional questionnaire was distributed that asked about oral health, dental and periodontal conditions, personal attitudes toward dental care, and smoking habits. Cross tabulation with chi-squared testing was carried out to investigate the association of toothbrush usage and smoking with several variables. Four-hundred and sixty subjects enrolled in the study. The majority of the respondents were students and Saudi males. More than 75% of the participants had neither a family dentist nor dental insurance; 7% were smokers, 84% used a toothbrush, 17% used dental floss and 34% used miswak (a teeth cleaning twig made from the Salvadora persica tree). Some of the individuals complained of tooth sensitivity, halitosis and bleeding gums. The main reason for dental visits was pain, with 23% of the participants having never visited a dentist. Tooth brushing was significantly associated with gender, nationality, occupation, education, marital status, having kids and dental insurance (*p* ≤ 0.05). Tobacco consumption was significantly associated with age, occupation, education level, marital status, having children, having bleeding gingivae and halitosis. Effective dental education programs are needed to improve dental knowledge and awareness in the Medina community.

## 1. Introduction

Medina is a multicultural city located in the center of western Saudi Arabia. In addition to Saudi citizens, there are residents who have different ethnic nationalities. This diversity of backgrounds can reflect on the community in general and oral health specifically. Dental health is one of the primary health measures that can affect quality of life. Dental and oral diseases can lead to and are associated with many systemic conditions, such as gastrointestinal problems, autoimmune disorders, cardiovascular diseases, speech difficulties, low self-esteem, psychological instability and diminished nutrition [1,2,3,4]. Otitis media/externa and asthma can also be closely correlated to periodontal diseases [5]. Additionally, serious health conditions such as stroke [6] and cancer risk [7] can be related significantly to poor oral hygiene.

Oral health can be affected by various factors, such as changes in lifestyle, limited access to dental care services, socio-economic status, education and personality [2,8,9]. The prevention of oral diseases can be ensured by following optimal oral hygiene habits, such as teeth brushing, using dental floss, avoiding smoking and regular dental visits [4,5]. Other important preventive factors are diet and nutrition, as these affect the development and progression of oral diseases and conditions [10,11].

Ensuring access to oral health care for all people regardless of other factors, such as socio-economic status, is critical to maintaining oral health [12]. A significant association has been found between education level and oral health; lower education levels correlate with a higher risk of dental health problems [13].

Though tobacco smoking (including passive smoking) is widely considered as one of three leading risk factors in the global disease burden [14], the number of people smoking in the Middle East in general, and in Medina in particular, is increasing [15,16,17,18]. Smoking is undoubtedly associated with poor oral health, along with behaviors such as frequent snacking and infrequent tooth brushing [14,19]. Smoking has a wide range of effects on the oral cavity, including tooth and restoration staining [20], increased susceptibility to periodontitis and reduced response to periodontal therapy, high risk of oral candidiasis, dental caries and dental implant failure [21], as well as high risk of oral pre-cancerous and cancerous lesions [20].

The evaluation of the oral health behavior of communities is fundamental to assess the general oral health and to aid in developing behavior modification and community education programs. Many studies have investigated oral health attitudes in the Medina population. However, they were limited to specific groups in the community. One study investigated the oral health practices and knowledge among the male administrative staff at Taibah University [22], whereas the populations of other studies were 9–12-year-old school children [23,24]. Further studies assessed the implementation of oral health knowledge and practices by mothers with their children [25] and the factors affecting oral health patterns among the female students at Taibah University [26]. The aim of the current study is to explore the oral health attitudes and behaviors in a random sample from the multicultural community residing in Medina.

## 2. Materials and Methods

### 2.1. Materials

The study was carried out in accordance with the Code of Ethics of the World Medical Association (Declaration of Helsinki). Ethical approval was obtained from the Research Ethics Committee of Taibah University, College of Dentistry (TUCODREC/20151122). A cross-sectional questionnaire was designed by one of the researchers and then revised by two other researchers. Validation of the questionnaire was done by running a pilot study that asked patients who were attending dental clinics at Taibah University to fill the questionnaire; collected data were cleaned. The pilot study was carried out on 10 subjects, twice, 10 days apart. Reliability of the questionnaire was measured with the Cronbach alpha coefficient. Its values were 0.87–0.91, indicating that the questionnaire had internal consistency.

The study was conducted on Oral Health International Day at the biggest mall in Medina, Saudi Arabia. The questionnaire was filled out by direct interviews with subjects who agreed to participate in the study. The inclusion criterion was a subject age of 18 years old or older, or permission of a parent/guardian for subjects younger than 18 years old. Participants signed an informed written consent form. The following information was obtained from the questionnaire: participants’ socio-demographics (age, gender, nationality, occupation, education level, marital status and number of children), oral health, smoking habits, dental and periodontal condition as well as personal attitude towards dental care.

### 2.2. Data Analysis

Data from the completed questionnaires were entered in the IBM-SPSS version 21 (Chicago, IL, USA), and statistical analysis was carried out. Basic descriptive information on socio-demographics and the oral health variables was obtained along with frequencies. Cross tabulation with chi-squared tests was also carried out to investigate the significance of the association of tooth brushing or smoking with various variables (age, gender, nationality, occupation, education, marital status, having dental insurance, etc.). Significance was set at *p* ≤ 0.05 and a 95% confidence interval.

## 3. Results

A total number of 475 individuals agreed to participate in the study. However, some of them did not provide answers to particular questions. All incomplete questionnaires were excluded from the study, resulting in a total of 460 subjects. The majority of participants were males (62%), Saudi (67%) and students (69%). Table 1 shows all the socio-demographic characteristics of the sample.

**Figure 1:**
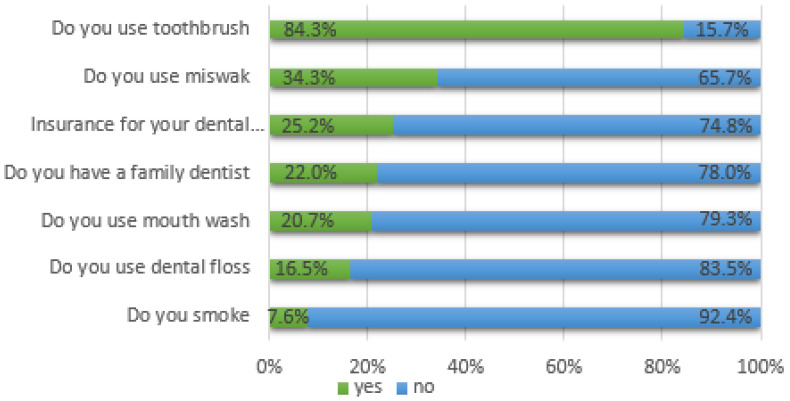
Oral-health-related characteristics of the study sample.

Oral-health-related attitudes and behaviors of the participants are displayed in Figure 1, which shows that 74.8% of the respondents did not have dental insurance and 78% did not have a family dentist. Only 7.6% admitted that they were smokers. A total of 84.3% of the participants used a toothbrush, but the percentages of the participants who used dental floss, mouth wash and miswak (a teeth-cleaning twig made from the Salvadora persica tree) were 16.5%, 20.7% and 34.3%, respectively.

**Figure 2:**
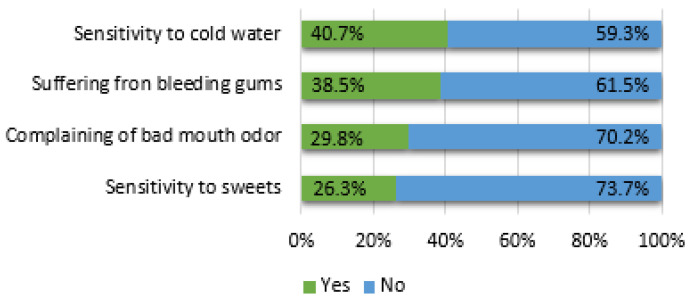
Dental and periodontal conditions.

A total of 26.3% of the participants complained of sensitivity to sweets, 40.7% had a cold sensitivity, 29.8% had self-perceived halitosis and 38.5% suffered from bleeding gums, as shown in Figure 2.

**Figure 3:**
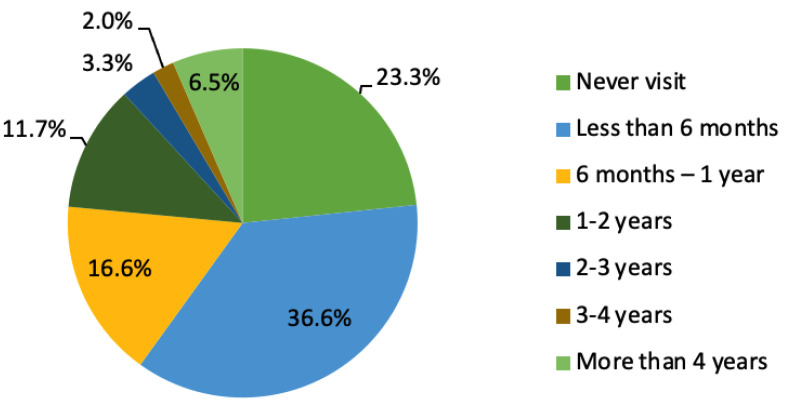
Participant dental visits.

Pain was the main reason to visit a dentist. A total of 74% of the participants visited the dentist to get rid of pain, 15% visited for a check-up, only 7% visited for aesthetic reasons and 4% had other reasons. The reasons for avoiding dental appointments included the following: 38% of respondents did not like going to the dentist, 29% denied having dental problems and 28% cited cost as the reason for avoiding dental appointments. Figure 3 shows the dental visit frequency of participants; 23% stated that they had never visited a dentist in their life, whereas the remaining participants had visited dental clinics at variable frequencies, ranging from less than six months to more than four years.

Table A1 shows the results of the cross tabulation of tooth brushing with other variables. There was a significant association between tooth brushing and the following variables: gender, nationality, occupation, education, marital status, having children and dental insurance. There was a significant association between tobacco consumption and age, occupation, education level, marital status, having children, bleeding gingivae and self-perceived halitosis, as shown in Table A2.

## 4. Discussion

The current study represents one of few studies that explore the oral health attitude in the general community of Medina. Results show a significant association between tooth brushing and female gender (*p* = 0.001), with 91% of female participants using a toothbrush. Many studies have confirmed that females exhibit better oral health attitudes than males [8,27].

The results of the cross tabulation of the use of toothbrushes with various factors show some interesting findings.

There was an abidance of at least 80% with the use of toothbrushes across various age groups; however, groups above 17 years of age showed higher use of toothbrushes (87.9–93.3%), indicating the need for more education in schools on the importance of using a toothbrush at an early age.

Non-Saudis used toothbrushes significantly more than Saudis (*p* = 0.015). This most likely reflects higher education levels of the foreign work force in this wealthy country. The use of toothbrushes was also significantly associated with the subject’s occupation, with the highest use (100%) coming from health care workers and the lowest from the retired group (*p* = 0.032). The use of toothbrushes was also significantly associated with education (*p* = 0.001), with the highest use reported by those with a bachelor’s degree and the lowest use again in preschool children, reflecting a significant finding in this study. Tooth brushing was also significantly associated with married/previously married people, as they showed higher use compared with single people (*p* = 0.001). Interestingly, those having dental insurance used toothbrushes significantly more often than those without insurance (*p* = 0.001). However, this can be explained on the basis that obtaining dental insurance reflects an already established interest in dental and oral health.

Although dental floss is an essential part of oral hygiene, only 17% of the participants disclosed using it. This result was expected because dental floss usage requires training and awareness. Many studies have shown that the percentage of medical and dental students as well as newly graduated dentists using dental floss is considered to be low [28,29].

Miswak was used by 34% of the participants as a traditional tooth cleaning aid. Miswak is used widely in developing countries due to cultural and religious background as well as to low cost and simplicity. Miswak has preventive and therapeutic effects on the oral cavity due to its wide range of antimicrobial [30], anti-fungal [31], anti-cariogenic/anti-plaque [32] and anti-inflammatory properties [33].

Despite the importance of regular dental visits, the results of the current study showed that approximately 47% of the participants were either irregular visitors or non-attendees, even though 41% of them complained of various dental symptoms (e.g., sensitivity to sweets, cold sensitivity, bleeding gingivae and halitosis). This result is in agreement with other studies carried out in Medina [22,23,25], Jeddah [34], Jordan [12,35] and Poland [1], and another study that investigated oral hygiene behavior among university students in 26 countries across Asia, Africa and the Americas [8], indicating the global need for more dental awareness. The main reason for dental visits in the current study was pain. This finding also coincides with other studies [12,22,26,27,34,35]. These two findings point to the need for the coordination of intensive dental education programs, in the community generally and in the schools specifically, focused on increasing awareness of regular dental visits and oral disease prevention. There is also a need for co-operation between ministries of health, dental schools and dental associations in order to educate and train health care providers in the promotion of oral hygiene. In addition, the reinforcement of regular intensive school-based oral health programs will lead to improved health literacy for children. Finally, social media can be used as an effective tool to promote oral hygiene motivation and education. Many studies have shown that patients are willing to learn from different social media channels; however, there are many challenges in ensuring that quality and evidence-based knowledge is delivered [36].

A significant number of the participants did not have dental insurance. Though free dental care services are available for Saudi citizens at government dental centers, there are usually waitlists that can delay the services offered to patients. On the other hand, non-Saudi residents attend private dental clinics to receive dental care, and this study showed that cost was the major factor in avoiding dental treatment. There is evidence that even when dental insurance was provided by employers, employees were not satisfied with dental insurance policies [37].

Generally, the prevalence of smoking in Saudi Arabia is high [20,22]. However, the percentage of smokers among the sample of the current study was 8%, which can be attributed to the fact that the sample consisted of both genders and various age groups (124 participants were 6–9 years old) as the data were collected from an event that was carried out in the biggest mall in the city, and events in such locations usually attract families with their children. The event was educational and included different activities. Commonly, the prevalence of smoking is significantly higher in men than in women [37,38]. Gender differences in smoking behavior are related to social factors, weight gain and family factors, in addition to the fact that women are less likely to smoke when they become mothers [39]. Moreover, smoking is prohibited in the holy city of Medina, and the selling of tobacco products is not allowed within Haram (boundaries of the old city of Medina). This explains the low smoking prevalence in this sample. It would be interesting to see what the effect would be on smoking rates if such a ban on tobacco products was also implemented in other cities. Smoking was significantly associated with age group, occupation, education, marital status, having children, having bleeding gums and having halitosis (*p*-values = <0.001, 0.001, 0.002, 0.001, <0.001, 0.046 and 0.032, respectively). The highest significant use of tobacco was in the 18–30-year-old age group, employees with jobs (probably reflecting their stress at work), post-graduate educated people, married/previously married people (probably again reflecting stress levels), those with children, and those reporting bleeding gums (it is difficult to determine cause and effect here) and having halitosis.

One-third of the participants complained of self-perceived halitosis. The majority of studies have reported the same percentage (30%) of halitosis [40], which is usually linked to periodontal or dental conditions. A total of 39% of participants were suffering from bleeding gingivae, indicating the presence of periodontium inflammation and contributing to the formation of volatile sulphur compounds and a greater prevalence of periodontal pathogens with a complex interaction between numerous oral bacteria species [41].

## 5. Conclusions

The results of the current study highlight the importance of the planning and coordination of effective intensive dental education programs for the community and at schools. Usage of miswak can be included in these programs. Some developing countries use miswak as part of their culture, and it is considered as an efficient oral hygiene tool. These programs will increase the awareness of dental health and encourage people to maintain regular dental visits instead of limiting visits to those for pain elimination, where the situation can become more complicated and can influence general health.

All variables in the current study had a significant relationship with tooth brushing and smoking. Dental insurance coverage should be considered as an integral component of health care for the entire population, regardless of nationality.

## Figures and Tables

**Table 1:** Socio-demographic characteristics of the sample.

Characteristics Mean ± SD	(*n*)	(%)
**Age group (19.0 ± 13.7 years)**		
4–9 (6.8 ± 1.6)	124	27.0
10–17 (12.4 ± 2.1)	155	33.7
18–30 (23.6 ± 3.8)	88	19.1
31–44 (35.9 ± 3.9)	60	13.0
45–72 (52.5 ± 7.3)	33	7.2
**Gender**		
Male	284	61.7
female	176	38.3
**Nationality**		
Saudi	310	67.4
Non-Saudi	150	32.6
**Occupation**		
Student or preschool	318	69.1
Employee	75	16.3
Retired	5	1.1
Health care employee	62	13.5
**Education degree**		
Pre-school	44	9.6
Primary school	201	43.7
Intermediate school	60	13.0
High school	62	13.5
Diploma	27	5.9
Bachelors	54	11.7
Postgraduate	12	2.6
**Marital status**		
Single	350	76.1
Married	105	22.8
Divorced	2	0.4
Widow(er)	3	0.7

## Appendix A

**Table A1:** Cross tabulation of toothbrush use and various variables.

Characteristics	Do You Use Toothbrush		*p*-Value
	Yes	No	
**Age group**			0.051
4–9 (*n* = 124)	100 (80.6%)	24 (19.4%)	
10–17 (*n* = 155)	124 (80.0%)	31 (20.0%)	
18–30 (*n* = 88)	79 (89.8%)	9 (10.2%)	
31–44 (*n* = 60)	56 (93.3%)	4 (6.7%)	
45–72 (*n* = 33)	29 (87.9%)	4 (12.1%)	
**Gender**			0.001
Male (*n* = 284)	227 (79.9%)	57 (20.1%)	
Female (*n* = 176)	161 (91.5%)	15 (8.5%)	
**Nationality**			0.015
Saudi (*n* = 310)	253 (81.6%)	57 (18.4%)	
Non-Saudi (*n* = 150)	133 (88.7%)	17 (11.3%)	
**Occupation**			0.032
Student/preschool (*n* = 318)	258 (81.1%)	60 (18.9%)	
Employee (*n* = 75)	71 (94.6%)	4 (4.6%)	
Health care employee (*n* = 62)	62 (100.0%)	0 (0.0%)	
Retired (*n* = 5)	3 (60%)	2 (40%)	
**Education**			0.001
Preschool (*n* = 44)	30 (68.2%)	14 (31.8%)	
School (*n* = 323)	271 (83.9%)	52 (16.1%)	
Diploma (*n* = 27)	23 (85.2%)	4 (14.8%)	
Bachelor (*n* = 54)	53 (98.1%)	1 (1.9%)	
Postgrad (*n* = 12)	11 (91.7%)	1 (8.3%)	
**Marital status**			0.001
Single (*n* = 350)	289 (82.6%)	61 (17.4%)	
Married/previously married (*n* = 110)	99 (90%)	11 (10%)	
**Smoking**			0.163
Yes (*n* = 35)	27 (77.1%)	8 (22.9%)	
No (*n* = 425)	361 (84.9%)	64 (15.1%)	
**Have dental insurance**			0.001
Yes (*n* = 116)	109 (94.0%)	7 (6.0%)	
No (*n* = 344)	279 (81.1%)	65 (18.9%)	
**Have bleeding gums**			0.104
Yes (*n* = 177)	144 (81.4%)	33 (18.6%)	
No (*n* = 283)	244 (86.2%)	39 (13.8%)	
**Have halitosis**			0.473
Yes (*n* = 137)	113 (82.5%)	24 (17.5%)	
No (*n* = 323)	275 (85.1%)	48 (14.9%)	

**Table A2:** Cross tabulation of tobacco use and various variables.

Characteristics	Do You Use Tobacco		*p*-Value
	Yes	No	
**Age group**			0.000
4–9 (*n* = 124)	0 (0.0%)	124 (100.0%)	
10–17 (*n* = 155)	3 (1.9%)	152 (98.1%)	
18–30 (*n* = 88)	16 (18.2%)	72 (81.8%)	
31–44 (*n* = 60)	12 (20.0%)	48 (80.0%)	
45–72 (*n* = 33)	4 (12.1%)	29 (87.9%)	
**Gender**			0.220
Male (*n* = 284)	25 (8.8%)	259 (91.2%)	
Female (*n* = 176)	10 (5.7%)	166 (94.3%)	
**Nationality**			0.907
Saudi (*n* = 310)	24 (7.7%)	286 (92.3%)	
Non-Saudi (*n* = 150)	11 (7.4%)	137 (92.6%)	
**Occupation**			0.001
Student (*n* = 318)	12 (3.8%)	306 (96.2%)	
Employee (*n* = 75)	24 (32%)	51 (68%)	
Retired (*n* = 5)	0 (0.0%)	5 (100.0%)	
Health care worker (*n* = 62)	8 (12.9%)	54 (87.1%)	
**Education**			0.002
Preschool (*n* = 44)	0 (0.0%)	44 (100.0%)	
School (*n* = 323)	20 (6.2%)	303 (93.8%)	
Diploma (*n* = 27)	3 (12.0%)	24 (88.0%)	
Bachelor (*n* = 54)	9 (11.2%)	45 (88.8%)	
Postgrad (*n* = 12)	3 (25.0%)	9 (75.0%)	
**Marital status**			0.001
Single (*n* = 355)	19 (5.4%)	336 (94.6%)	
Married/previously married (*n* = 105)	16 (15.2%)	89 (84.8%)	
**Have children**			0.000
No (*n* = 358)	19 (5.3%)	339 (94.7%)	
Yes (*n* = 102)	16 (15.7%)	86 (84.3%)	
**Have dental insurance**			0.944
Yes (*n* = 116)	9 (7.8%)	107 (92.2%)	
No (*n* = 344)	26 (7.6%)	318 (92.4%)	
**Have bleeding gums**			0.046
Yes (*n* = 177)	19 (10.7%)	158 (89.3%)	
No (*n* = 283)	16 (5.7%)	267 (94.3%)	
**Have halitosis**			0.032
Yes (*n* = 137)	16 (11.7%)	121 (88.3%)	
No (*n* = 323)	19 (5.9%)	304 (94.1%)	
